# Supplementary material for: Venomix: a simple bioinformatic pipeline for identifying and characterizing toxin gene candidates from transcriptomic data
Source: PeerJ. 2018 Jul 31;6:e5361. doi: 10.7717/peerj.5361 (PMC6074769; doi:10.7717/peerj.5361)
Supplement: Supplemental Information 3 [file peerj-06-5361-s003.gz › FinalOutput_E-20/Zinc_metalloproteinase-disintegrin-like_alternagin_2/finaltree.pdf]

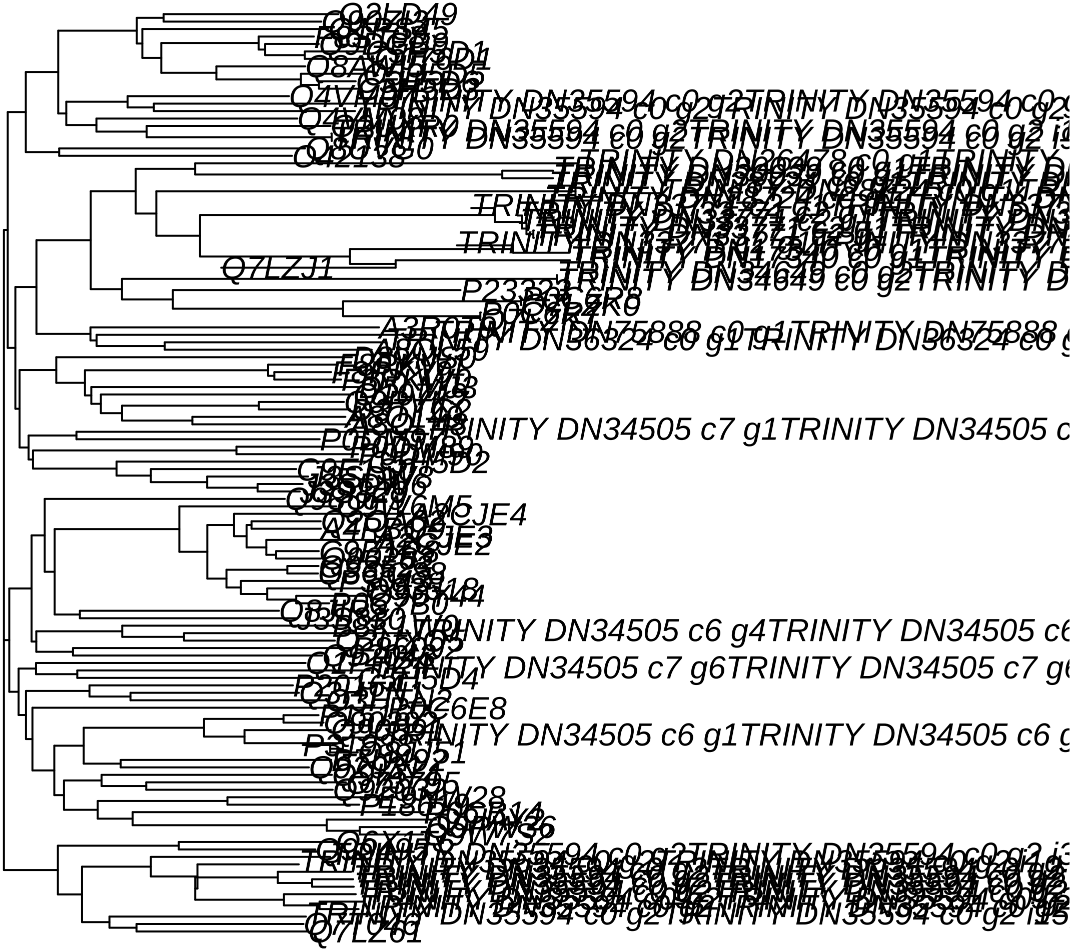
Q2LD49
Q7LZJ1
TRINITY DN34649 c0 g2TRINITY D
TRINITY DN34505 c7 g1TRINITY DN34505 c
TRINITY DN34505 c6 g4TRINITY DN34505 c6
TRINITY DN34505 c7 g6TRINITY DN34505 c7 g
TRINITY DN34505 c6 g1TRINITY DN34505 c6 g
Q7LZ61
